# Chronic Pain in Multiple Sclerosis: Mechanisms, Clinical Characteristics and Treatment Strategies

**DOI:** 10.3390/ijms27020873

**Published:** 2026-01-15

**Authors:** Panagiotis Gklinos, Georgia Christodoulou, Dorothea Pournara, Maria-Eleftheria Evangelopoulos, Dimos-Dimitrios Mitsikostas

**Affiliations:** 1st Department of Neurology, Eginition University Hospital, National and Kapodistrian University of Athens, 11528 Athens, Greece

**Keywords:** multiple sclerosis, pain, chronic pain, pathogenesis, treatment, review

## Abstract

Chronic pain is an underestimated and undertreated yet highly prevalent symptom in people with multiple sclerosis (pwMS), significantly impairing quality of life and functional outcomes. Its prevalence ranges from 23% to 90% across studies, reflecting methodological differences and discrepancies in the definition and recognition of chronic pain. In this article, we aim to provide an updated review of the pathophysiological mechanisms of chronic pain in MS, including the effect and interaction between neuropathic, nociceptive and nociplastic mechanisms, and propose a mechanism-based classification. Furthermore, we explore different therapeutic approaches, including both pharmacological and non-pharmacological interventions, tailored to each patient according to the mechanism involved. A deeper understanding of the distinct chronic pain mechanisms and phenotypes can provide more effective and personalized treatment strategies and lead to improved patient outcomes and quality of life.

## 1. Introduction

Chronic neurological conditions are a major contributor of disability among people worldwide, with significant socioeconomic impact. Multiple sclerosis (MS) is a chronic, immune-mediated disease of the central nervous system (CNS), characterized by demyelination, inflammation and axonal loss, resulting in a broad spectrum of neurological symptoms and disability. MS typically begins between the third and fourth decade of life, affecting about 2.5 million individuals worldwide with a threefold prevalence in females. It is considered among the most common causes of non-traumatic neurological disability in young adults [1]. While the exact pathogenesis of MS is yet to be fully understood, genetic, immune and environmental factors are known to be involved [1]. Although the disease primarily manifests with either motor, sensory or visual symptoms, pain is one of the most commonly reported symptoms by people with MS (pwMS) and it is known to significantly affect their quality of life. Pain may be the results of central and peripheral mechanisms, reflecting the heterogeneous biology of the disease. Chronic pain is defined by the International Association for the Study of Pain (IASP) as pain that persists or recurs for longer than 3 months [2]. According to IASP, chronic pain has a significant impact on physical, psychological as well as social aspects, and its management can be complex, as it often requires addressing both its biological and psychosocial contributors. A comprehensive understanding of these components is crucial in order to identify and apply individualized, patient-centered strategies to improve pain management and ultimately quality of life of pwMS.

## 2. Literature Search and Study Selection

This study represents a comprehensive review of chronic pain in MS. Studies were identified after search in PubMed and related bibliographic databases, focusing on studies reporting epidemiology, pathophysiology, mechanisms, clinical presentation and treatment (pharmacological and non-pharmacological) of chronic pain in pwMS. Original peer-reviewed research studies, including observations and clinical trials, as well as systematic reviews, meta-analyses and consensus statements, were included in our review. Priority was given to clinically relevant studies and mechanistically informative studies. Studies were excluded if they focused on acute pain, non-MS or pediatric populations and pain conditions not related to central nervous system pathology.

## 3. Epidemiology

The reported prevalence of pain in MS patients varies widely, ranging from 29% to 86% across studies [3,4,5,6,7,8,9,10,11,12], with a pooled prevalence of 63% (17 studies, 5319 participants; 95% confidence interval (CI) = 55.1–70.3%) [13]. This wide range reflects differences in study designs as well as discrepancies in definitions and recognition of pain in pwMS [14]. A meta-analysis of 28 articles (7101 subjects) [13] reported that the most frequent form of pain was headache (43%), followed by neuropathic extremity pain (26%), back pain (20%), painful spasms (15%), Lhermitte’s sign (16%) and trigeminal neuralgia (3.8%). In a multicenter cross-sectional study, Solaro et al. [9] evaluated pain types in pwMS, including dysesthetic pain, back pain, painful tonic spasms, Lhermitte’s sign, visceral pain and trigeminal neuralgia. They demonstrated that pain was associated with disability, age, disease duration and disease course, but not with sex, except for trigeminal neuralgia, where statistical analysis revealed a significant difference on every measure. In contrast, visceral pain was correlated only with gender and not other variables [9]. Increased pain intensity was positively associated with female sex, increased age, greater duration of pain and increased MS-related disability [11]. Additionally, Obsorne et al. [15] found that psychosocial factors, such as pain-related catastrophizing, social support, pain beliefs and pain coping, were strongly associated with increased pain intensity and pain interference, with catastrophizing having the most consistent connection. In a case-control study by Svendsen et al. [16], although the authors reported a high prevalence of pain both in MS patients and controls (79.4% in MS patients vs. 74.7%, respectively), MS patients had a higher pain intensity, higher need for analgesic treatment and greater impact of pain on daily activities. When comparing pain prevalence in different courses of MS, it was found that the pooled prevalence was 50% in relapsing remitting MS (5 studies, 2089 subjects with RRMS), 69.8% in secondary progressive MS (5 studies, 673 patients with SPMS) and 70.3% in primary progressive MS (5 studies, 393 patients with PPMS) [13]. Furthermore, a recent meta-analysis reported increased nocebo phenomena in pwMS, including pain-related symptoms [17]. More specifically, the pooled prevalence of nocebo responses was 83%, with headache and back pain being the most frequently reported pain syndromes, reported by 8–33% and 2–11% of patients, respectively. Besides nocebo phenomena, according to the same study, pwMS who were on the active arms of the trials also experienced various types of pain syndromes, such as back pain (8–11%), headache (5–44%) or other musculoskeletal disorders (44%) [17].

## 4. Classification of Pain and Pathophysiology

**Figure 1:**
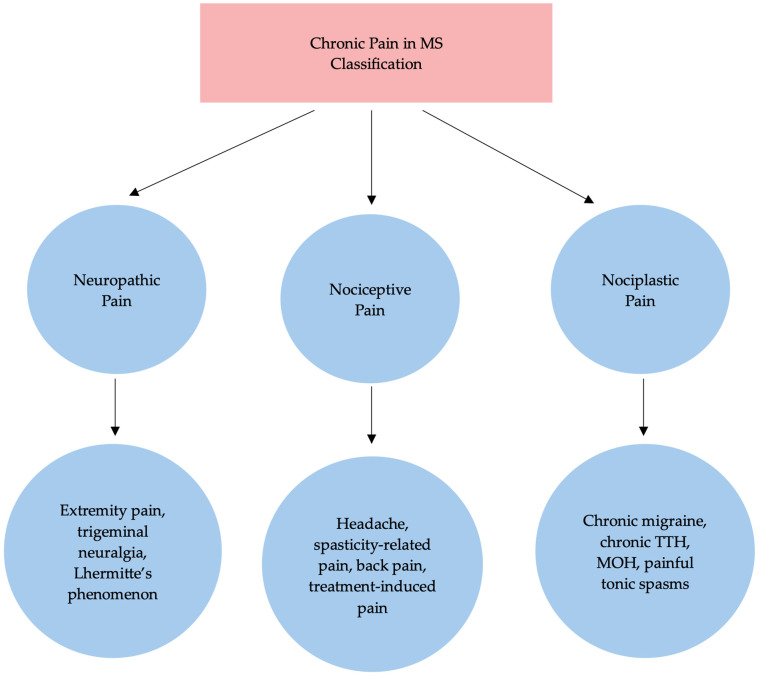
Proposed classification of chronic pain in MS. TTH: tension-type headache; MOH medication overuse headache.

Given the high prevalence and clinical relevance of pain in MS, several classification systems have been proposed to better understand its mechanisms and guide treatment. Pain in MS has been classified based on different characteristics, such as intensity, location or duration. O’Connor et al. proposed a classification based on its pathophysiology, which Truini et al. later recreated, dividing pain syndromes experienced by pwMS essentially into three main categories: neuropathic, nociceptive and nociplastic pain [4,18]. However, emerging molecular evidence has demonstrated that there is a substantial mechanistic overlap between these categories, particularly in chronic conditions. Nociceptive pain refers to pain caused by damage or dysfunction of non-nervous tissue and results from the activation of nociceptors (e.g., the vanilloid receptor TRPV1), whereas neuropathic pain originates from damage or dysfunction of the somatosensory nervous system [19]. Nociplastic pain reflects pain that results from altered nociception without clear evidence of tissue injury and involves peripheral or central sensitization [18]. Our proposed mechanism-based classification of chronic pain, linking clinical phenotypes to structural lesions and molecular pathways, is illustrated in Figure 1.

### 4.1. Neuropathic Pain

Neuropathic pain results from direct damage or dysfunction of the somatosensory system. Beyond demyelination, it also reflects ion channel alterations, glial activation and disrupted inhibitory control, leading to spontaneous ectopic discharges, hyperexcitability and allodynia, hence occasionally overlapping with nociplastic mechanisms [18]. Common molecular mechanisms include ectopic axonal activity resulting from the redistribution and overexpression of voltage-gated sodium channels—particularly Nav1.6—at demyelinated segments, which facilitates aberrant high-frequency firing and ephaptic transmission. In addition, microglial activation via P2X4/P2X7 and Toll-like receptor 4 (TLR4)-dependent pathways potentially lead to the release of proinflammatory mediators, including tumor necrosis factor-α (TNF-α), interleukin-1β (IL-1β) and interleukin-6 (IL-6), as well as brain-derived neurotrophic factor (BDNF). BDNF–tropomyosin receptor kinase B (TrkB) signaling downregulates potassium-chloride cotransporter 2 (KCC2), shifting GABAergic transmission to depolarization and thus contributing to central disinhibition [4]. Furthermore, astrocytic dysfunction, and particularly reduced glutamate transporter 1 (GLT-1) expression, may result in excessive extracellular glutamate and increased N-methyl-D-aspartate (NMDA) and α-amino-3-hydroxy-5-methyl-4-isoxazolepropionic acid (AMPA) receptor activation [4]. Finally, lesions within the PAG can potentially disrupt the inhibitory, antinociceptive signaling at the level of the brainstem, hence resulting in central sensitization and increase in pain intensity [18].

Regarding specific phenotypes—further classified as continuous or intermittent in O’Connor’s framework—the most common syndromes include extremity pain, trigeminal or other neuralgias and Lhermitte’s phenomenon [4,18].

#### 4.1.1. Extremity Pain

The MS-specific phenomenon of constant, burning pain, exacerbated by physical activity and at nighttime, affecting the feet and legs bilaterally, was described by O’Connor et al. [4] as dysesthetic extremity pain and by Truini et al. as ongoing extremity pain [20]. It is the most common type of pain in MS and is associated with higher disability levels [21]. It is generally attributed to demyelination of the spino-thalamo-cortical pathways given its distal and bilateral distribution, which is also widely supported by MRI findings [22]. However, the pathophysiology of extremity pain appears to be more complex, with studies suggesting involvement of the dorsal root entry zone [23], periventricular white matter and microglial activation in the dorsal horn [24]. Except for the structural disconnection, the upregulation of calcium channel, voltage-dependent and α2δ-1 (Cavα2δ-1) subunits in dorsal horn terminals and microglial P2X4 to BDNF signaling promote neuronal hyperexcitability and tactile allodynia. At the same time, astrocytic GLT-1 downregulation, as mentioned before, further amplifies glutamate-mediated excitability. These mechanisms provide the foundation for various treatment modalities, including gabapentinoids, antidepressants, cannabinoids and neuromodulation.

#### 4.1.2. Trigeminal Neuralgia

MS-related trigeminal neuralgia (TN), clinically similar to idiopathic TN, is characterized by episodes of paroxysmal facial pain, described as electric-like, which can occur spontaneously or be triggered by external stimuli. Unlike idiopathic TN, MS-related TN is often bilateral and can affect younger patients and the eye [25]. The pathophysiology of MS-related TN is explained by two main hypotheses: ephaptic transmission between neighboring demyelinated axons creating discharges in both directions and mechanical compression of the trigeminal root [26,27]. According to Truini et al., these processes may coexist in MS, where autoimmune demyelination at the trigeminal root entry zone and mechanical demyelination due to neurovascular contact act synergistically to produce this pain syndrome [18]. These hypotheses have been supported by findings from neuroimaging and neurosurgical studies conducted by various researchers [27,28,29,30,31]. Finally, microglial activation within the trigeminal nuclei and altered GABAergic signaling may also contribute to attack pathogenesis as well as chronification.

#### 4.1.3. Lhermitte’s Phenomenon

Lhermitte’s phenomenon, often experienced by pwMS, is described as an abrupt, transient electric-like sensation most frequently in the nuchal region and lower back, triggered by neck flexion. In most cases, Lhermitte’s phenomenon lasts only a few weeks and resolves without any treatment [18,32]. This phenomenon is considered to have a similar pathophysiology to that of TN due to their similar quality and duration of pain, with the demyelination being located in the ascending spinothalamic tracts of the cervical area, as seen in MRI and postmortem studies [32,33].

### 4.2. Nociceptive Pain

Nociceptive pain reflects the activation of peripheral nociceptors due to damage or dysfunction of non-nervous tissue. At a molecular level, it involves transient receptor potential vanilloid 1 (TRPV1), transient receptor potential ankyrin 1 (TRPA1) and acid-sensing ion channels (ASICs) on peripheral nerve endings, the release of prostaglandin E2 (PGE2) via cyclooxygenase-2 (COX-2), substance P, calcitonin gene-related peptide (CGRP) and inflammatory cytokines as well as secondary dorsal horn plasticity when stimulation becomes chronic [4,18].

#### 4.2.1. Headache

Headaches are a common symptom in pwMS, with migraines being the predominant type, experienced by approximately one-third of pwMS. More specifically, recent studies, including a two-year prospective study, have shown that pwMS are more than two times more likely to suffer from migraines compared to the general population, while the prevalence of tension-type headache also appears to be increased in pwMS, but not as much as migraine [34,35,36]. Besides frequency, pwMS tend to experience more disabling headaches, which affect quality of life and lead to an increased use of analgesic medication [34]. The occurrence of migraine in MS has been linked to several theories, with the most predominant one being the presence of demyelinating lesions across areas important for pain transmission and perception [37]. More specifically, demyelination and subsequent axonal loss within these regions activate the pathophysiological cascades of migraine, probably through the disruption of inhibitory pain pathways such as through demyelination of periaqueductal gray (PAG). Other theories include immune system aspects such as the expression of calcitonin gene-related peptide (CGRP) by activated lymphocytes as well as the demyelination of cortical neurons, which can potentially trigger aura through enhancing aberrant excitability of the cortex, leading to cortical spreading depression, the backbone of migraine with aura [38].

#### 4.2.2. Treatment-Induced Pain

Pain related to treatment should not be overlooked, as it also plays an important role in the quality of life of pwMS. Treatments commonly associated with secondary pain include interferon-beta and corticosteroids, with manifestations ranging from pain in the injection site to myalgias and headaches [4].

#### 4.2.3. Spasticity-Related Pain

Pain secondary to spasticity, not to be confused with the tonic spasms described below, is related to the abnormally increased muscle tone resulting from damage to central motor pathways involved in voluntary movement. It typically presents with muscle stiffness, painful contractions and limited mobility, often aggravating mobility difficulties [33,39]. Sustained muscle contractions trigger the release of several inflammatory mediators, including PGE2, TNF-α, IL-1β and IL-6, which further sensitize peripheral nociceptors.

#### 4.2.4. Back Pain

Frequently observed in pwMS, low back pain is considered to be mostly secondary to incorrect posture, prolonged immobilization and improper wheelchair and mobility aid use, and less related to mechanisms related to MS itself [4].

### 4.3. Nociplastic or Mixed Pain

Nociplastic pain stems from altered nociceptive processing, mainly due to the persistent overactivation and dysfunction of pain-related CNS structures such as the thalamus, PAG or spinal trigeminal nucleus, resulting in allodynia and central sensitization. Failure of serotonergic and noradrenergic descending inhibition from the PAG and rostral ventromedial medulla is prominent in nociplastic conditions, while other molecular pathways include functional reorganization within thalamic and cortical pain networks, and persistent microglia activation and astrocytic cytokine release [4,18].

#### 4.3.1. Painful Tonic Spasms

Painful tonic spasms are an MS-specific phenomenon that includes brief, involuntary contraction of the muscles, most commonly of the lower or upper extremities, as a response to sensory stimuli or movement. The spasms last for a few seconds and may occur multiple times per day over several weeks or months [40]. According to MRI studies, the origin of painful tonic spasms lies in lesions within the corticospinal pathways, particularly in the internal capsule, cerebral peduncle and spinal cord [40]. The underlying mechanism is thought to involve ectopic high-frequency discharges in demyelinated motor fibers, leading to hyperexcitability and synchronous activation of adjacent motor units, which explains their paroxysmal character [4,40].

#### 4.3.2. Chronic Migraine, Chronic TTH and Medication Overuse Headache (MOH)

According to O’Connor et al., migraine and headache disorders are more appropriately classified as nociplastic or mixed pain conditions, whereas Truini et al. have categorized headache within the nociceptive pain domain [4,18]. In fact, while episodic headache clearly represents a nociceptive process, chronic migraine, chronic TTH and MOH all involve central sensitization mechanisms, including altered pain modulation, lower pain thresholds and dysfunction of critical, pain-related brain structures such as the thalamus, PAG and dorsal raphe nucleus (DRN). Consequently, they may be more appropriately classified as nociplastic rather than nociceptive, or may represent an overlap between the two mechanisms.

## 5. Treatment

### 5.1. Pharmacological Treatment

The following section includes a summary of the available pharmacological treatments for chronic pain in MS. Dose ranges are summarized in Table 1, while routes of administration are specified in the text; treatment duration is reported selectively where supported by consistent trial data.

#### 5.1.1. Anticonvulsant Drugs

Anticonvulsant drugs are widely used in treating neuropathic pain associated with multiple sclerosis. They are given either orally or parenterally. Benzodiazepines, such as diazepam and clonazepam, enhance the effect of GABA and suppress neuronal activity in CNS, contributing to muscle relaxation, hence representing a promising option in the treatment of spasticity-related chronic pain. In a randomized placebo-controlled study comparing clonazepam to baclofen and placebo over a period of 5 days to 20 weeks [41], clonazepam was significantly more effective than placebo in reducing spasticity and showed similar efficacy with baclofen. Diazepam was also compared to baclofen in some smaller trials [42,43]. Both diazepam and baclofen improved spasticity, with no significant differences between them. Moreover, according to a systematic review for the pharmacological management of spasticity in MS patients [44], diazepam produced a similar reduction in spasticity when compared with tizanidine and dantrolene. Because of their action in reticular formation, sedation is a common adverse effect. However, a potential benefit of the sedation could be associated with an antispastic effect at night, providing uninterrupted sleep [45]. Other adverse effects of benzodiazepines include incoordination, imbalance, drowsiness and lower extremity muscle weakness at high doses [46].

Gabapentin is an anticonvulsant drug which displays efficacy in the management of neuropathic pain and is administered orally [47]. Gabapentin binds to the α2-δ protein of voltage-gated calcium channels, inhibiting calcium entry and the release of excitatory neurotransmitters in the CNS [48]. An open-label study [49] assessed the efficacy of gabapentin in relieving pain in multiple sclerosis, with 31.8% of the MS patients reporting excellent pain relief and 36.3% reporting medium pain relief with gabapentin at an average daily dose of 600 mg. Moreover, two randomized placebo-controlled trials reported a statistically significant improvement in spasticity in the gabapentin-treated patients when compared to placebo. Gabapentin in doses of up to 900 mg orally three times a day over a 6-day period in the first study [50], and 400 mg orally three times a day for 48 h in the second study [51], decreased the Ashworth scale in MS patients. Duration of treatment varied across studies, with a median time of treatment of 86 days in one of them [47]. In clinical practice, the normal starting dose is 300 mg per day, escalated up to a maximum daily dose of 3600 mg [44]. The most common adverse effects during gabapentin therapy are drowsiness, somnolence, dizziness, fainting, ataxia, nystagmus, tremor and headache [44,45,48].

Pregabalin is also used for the treatment of neuropathic pain. According to its mechanism of action, pregabalin binds to the α2-δ subunit of calcium channels, reducing calcium influx and the release of neurotransmitters. This action leads to the prevention of seizure activity, as well as to analgesic and anxiolytic effects [52]. Pregabalin is given orally. According to a meta-analysis of randomized clinical trials, treatment duration varies from 5 to 20 weeks (median 8) [53]. Although several studies have demonstrated the benefits of pregabalin in various neuropathic pain conditions, such as diabetic peripheral neuropathy, postherpetic neuralgia, poststroke pain and spinal cord injury-related pain [53], trials for its use in pain associated with MS specifically are limited. An open-label study evaluated the efficacy of pregabalin (mean dosage of 154 mg/day) in 16 MS patients with paroxysmal painful symptoms. In this study, pregabalin improved pain symptoms in nine out of sixteen patients within one month of treatment [54]. Moreover, in an open-label study, pregabalin in combination with lamotrigine successfully treated trigeminal neuralgia in patients with multiple sclerosis [55]. Common adverse events associated to pregabalin treatment are weight gain, somnolence, dizziness, peripheral oedema, fatigue, visual disturbances, ataxia, vertigo and euphoria [52,53].

Carbamazepine blocks sodium channels, thereby reducing synaptic transmission. It is a first-line treatment for trigeminal neuralgia, which constitutes about 3.8% of pain syndromes in multiple sclerosis. It is administered orally. However, well-designed controlled trials evaluating its efficacy in multiple sclerosis-related pain are lacking. In a study of 33 MS patients with trigeminal neuralgia, carbamazepine provided complete or partial pain relief in 75% of the patients [56]. In a systematic review of the management of trigeminal neuralgia secondary to multiple sclerosis, the authors did not support a specific medical therapy due to insufficient evidence; however, as for the pharmacological treatment, they concluded that carbamazepine can be used as a first-line treatment for non-MS patients with trigeminal neuralgia [57]. Moreover, a case reported four MS patients with glossopharyngeal neuralgia, three of whom successfully responded to carbamazepine [58]. On the other hand, Ramsaranisng et al. reported five patients with multiple sclerosis whose disability significantly increased when using carbamazepine [59]. Patients under carbamazepine therapy may experience dizziness, drowsiness, ataxia, nausea, vomiting, skin rash, hypertension, bradycardia, neutropenia and abnormal liver function [60,61]. Liver function should be closely monitored in pwMS, as specific DMTs may also cause liver damage, including dimethyl-fumarate or fingolimod.

Lamotrigine is an oral antiepileptic drug that acts as a voltage-gated sodium channel inhibitor, decreasing the release of glutamate and aspartate. It has been shown that lamotrigine may reduce various forms of neuropathic pain, including post-stroke pain, diabetic neuropathy and trigeminal neuralgia, at doses of 300–400 mg per day [62]. According to a systematic review by Zakrzewska et al., lamotrigine may be considered as a second-line treatment for trigeminal neuralgia in patients with multiple sclerosis [57]. Treatment duration varied, with one of the trials reporting 8 weeks of treatment [57]. However, in a randomized, double-blind, placebo-controlled trial involving 12 MS patients with central pain, lamotrigine (up to 400 mg daily) did not demonstrate a statistically significant reduction in mean pain intensity when compared to placebo [63]. It is important to note that lamotrigine is associated with a risk of life-threatening reactions, such as Stevens–Johnson Syndrome, Drug Reaction with Eosinophilia and Systemic Symptoms (DRESS) syndrome and Toxic Epidermal Necrolysis (TEN) [61,62,64]. Additional adverse events include nausea, vomiting, drowsiness, dizziness, headache, visual disturbances, anxiety, dry mouth, weight loss and, in rare cases, cardiovascular and hematologic abnormalities [61,62,64].

Levetiracetam is an oral or parenteral anticonvulsant drug that modulates the release of neurotransmitters through binding to a synaptic vehicle protein 2A (SV2A). In a pilot, randomized, placebo-controlled study enrolling 20 patients with central neuropathic pain due to multiple sclerosis, levetiracetam, in daily doses of 2000–3000 mg, significantly improved pain-related study outcomes compared to placebo for a treatment period of 3 months [65]. Also, in a case series of 12 patients with multiple sclerosis [66], levetiracetam reduced phasic spasticity, measured by the Penn Spasm score, but had no effects on tonic spasticity, measured by Ashworth scores. In contrast, Falah et al., in a randomized, double-blind, placebo-controlled study of MS patients with central pain treated with 3000 mg of levetiracetam per day, reported no significant differences in pain relief and total pain intensity between levetiracetam and placebo within 6 weeks of treatment [67]. However, a higher reduction in pain intensity was demonstrated in subgroups with specific pain symptoms when treated with levetiracetam [67]. Finally, a systematic review and meta-analysis evaluated the efficacy of levetiracetam in neuropathic pain of different etiologies across six studies, including the previous study by Falah et al., and reported no benefits of levetiracetam in reducing neuropathic pain [68]. The most common adverse effects of levetiracetam are neurobehavioral, such as sedation, agitation, aggression, depression, fatigue, headache and dizziness, as well as nasopharyngitis, nausea and vomiting. More serious but rare side effects include life-threatening hypersensitivity reactions (SJS, DRESS and TEN) and suicidal thoughts [61,69]. Caution is warranted in pwMS with comorbid depression or other behavioral/psychological symptoms.

Topiramate is an oral antiepileptic drug with many off-label uses, including treatment of neuropathic pain. Topiramate has numerous pharmacological targets, such as voltage-gated sodium channels, AMPA/Kainate receptors, high-voltage-activated calcium channels, GABA-A receptors and carbonic anhydrase isoenzymes [70]. The evidence regarding its efficacy in MS-related pain is extremely limited. Treatment with topiramate led to the complete resolution of pain in six MS patients with refractory trigeminal neuralgia [71]. In this report, topiramate was used as a monotherapy in five patients and in combination with carbamazepine in the sixth patient at daily doses of 200 mg and 300 mg, respectively. In another case report, topiramate at a daily dose of 150 mg successfully treated dysesthetic pain of lower limbs in a patient with multiple sclerosis within 8 months of treatment [72]. Additionally, in a small open-label study enrolling MS patients with painful paroxysmal symptoms, topiramate (mean dose of 340 mg/day) significantly reduced the VAS score in 77% of the participants after 12 weeks of treatment [73]. The most common adverse effects include dizziness, somnolence, nausea, fatigue, paresthesia and weight loss [61,70]. Topiramate can also cause cognitive dysfunction, including word-finding difficulties, and should be used with caution in pwMS who may already experience cognitive deficits.

#### 5.1.2. Antidepressants

Duloxetin is an oral serotonin–norepinephrine reuptake inhibitor that can provide relief of neuropathic pain [74,75]. In a systematic review and meta-analysis of 10 studies by Finnerup et al. [74], the authors found that the NNT for 50% pain relief was 6.4 (95% CI, 5.2–8.4) for serotonin–noradrenaline reuptake inhibitor antidepressants. Two randomized placebo-controlled trials have proven the effectiveness of duloxetine in neuropathic pain associated to multiple sclerosis. In a study involving 239 patients with neuropathic pain [76], duloxetine-treated patients (taking 30 mg for 1 week and then 60 mg for 5 weeks) had a statistically greater mean improvement in average pain intensity vs. the placebo group at week 6 (−1.83 vs. −1.07, *p* = 0.001), and the number needed to treat (NNT) was 8. In a second study, the authors reported a reduction in average daily pain of 39% (±29%) in the duloxetine group compared to 10% (±18.8%) in the placebo group (*p* = 0.002) [77]. The most common side effects of duloxetine identified in clinical trials are dizziness, somnolence, nausea, dry mouth, fatigue, constipation, urinary retention, decreased appetite, insomnia, hypertension and transient and permanent sexual dysfunction [61,76,77,78].

Tricyclic antidepressants are a potential therapeutic option for neuropathic pain [74,75]. According to their mechanism of action, TCAs increase serotoninergic and noradrenergic transmission at the synaptic level by inhibiting the reuptake of neurotransmitters, and also block alpha-adrenergic, serotonergic, histamine and muscarinic receptors [79]. Across 15 studies [74], the NNT for 50% neuropathic pain relief was 3.6 (95% CI, 3.0–4.4) for tricyclic antidepressants, including amitriptyline, nortriptyline, maprotiline, imipramine and desipramine, making them an effective oral option. However, they should be offered with caution to pwMS, as they can potentially negatively affect cognition. Only one randomized controlled trial studied the efficacy of tricyclic antidepressants in MS patients with pain, comparing nortriptyline to transcutaneous electrical nerve stimulation [80]. The study showed a significant decrease in pain and sensory complaints of the upper extremities in both groups at 8 weeks, with no significant difference between them. The VAS score for the nortriptyline-treated group (10 mg daily increase over a week to 50 mg) improved from 4.9 ± 1.9 to 3.3 ± 2.1 (*p* < 0.001). Side effects of TCAs include dizziness, dry mouth, constipation, urinary retention, tachycardia, orthostatic hypotension, sedation, increased appetite, weight gain, blurred vision and confusion in patients [79,81].

#### 5.1.3. Muscle Relaxants

Tizanidine is a short-acting muscle relaxant that has been shown to be effective against MS spasticity, hence making an alternative option for spasticity-related pain in pwMS [44,82]. It is administered through the oral route. As a central α2 adrenoceptor agonist, it reduces the release of excitatory neurotransmitters at the spinal and supraspinal levels. Two randomized, double-blind, placebo-controlled trials in the US [83] for 15 weeks and in the UK [84] evaluated tizanidine as a treatment for spasticity in MS patients compared to placebo. The UK study reported a significant decrease in spastic muscle tone with tizanidine, while the US study found no significant differences in Ashworth scores. Moreover, in a double-blind, placebo-controlled trial, tizanidine reduced clinical spasticity and hyperreflexia in MS patients, with no change in functional status [85]. A multicenter double-blind, placebo-controlled, dose–response (8 or 16 mg of tizanidine) trial including 142 patients confirmed the antispasticity effect of tizanidine, and noted that both therapeutic and side effects are dose-related [86]. Tizanidine is generally well tolerated [87,88], though side effects include somnolence, fatigue, dry mouth, dizziness and hypotension [44,86,88,89]. Sublingual tizanidine has shown improvement in next-day spasticity compared with placebo without increasing next-day somnolence [90].

Baclofen is also a therapeutic option for MS spasticity and can potentially contribute to reducing spasticity-related chronic pain in pwMS. It can be administered through the oral or parenteral rout as an injection or intrathecally. Acting as a pre- and postsynaptic GABA-B agonist at the spinal level, it reduces the release of excitatory neurotransmitters, leading to the inhibition of mono- and polysynaptic reflexes at the spinal cord [91]. Several trials have demonstrated the effectiveness of baclofen on the improvement of spasticity-related symptoms, such as spasms, pain, stiffness and resistance to passive joint movements, associated with multiple sclerosis [92,93,94,95]. In a systematic review for the pharmacological treatment of spasticity in MS, the authors found a statistically significant improvement on spasticity with baclofen when compared to placebo in six out of the seven placebo-controlled trials that were assessed [44]. The most common side effects associated to oral baclofen are drowsiness, dizziness, weakness, dry mouth, headache, nausea, trouble sleeping, increased urination and constipation [44,61].

Intrathecal baclofen is an alternative therapy for patients with severe spasticity in whom oral antispasmodics have failed or the side effects from oral baclofen are intolerable [96,97,98]. The direct infusion of drug into the subarachnoid space achieves much higher concentrations in the cerebrospinal fluid, while the central side effects of oral baclofen, such as drowsiness or confusion, are minimized [99]. Several studies have examined the effectiveness of ITB treatment on patients with severe spasticity, including MS patients, and concluded that intrathecal baclofen is a safe and effective therapeutic option [97,100,101,102,103,104]. In a recent systematic review across seven studies evaluating the effectiveness of intrathecal baclofen in the treatment of MS spasticity [96], the authors found an average percent reduction of 61.25% on the Ashworth scale from pre- to post-ITB implantation, and noted that most complications were surgical (catheter dysfunction and misplacement, pump malfunction and infections) rather than pharmacological (headache, respiratory distress from overdosing, worsening cognitive functions, nausea, dizziness and drowsiness).

Dantrolene is a peripherally acting oral muscle relaxant that has been shown to be effective against MS spasticity [44,105]. Dantrolene acts directly on skeletal muscles, inhibiting the contractile mechanism. In a systematic review by Otero-Romero et al., the authors reported that dantrolene was superior compared to placebo in reducing spasticity associated to multiple sclerosis; however, their findings were based on low-quality evidence [44]. According to this review [44], dantrolene is recommended only in patients where no clinical improvement is seen with oral baclofen, tizanidine or gabapentin. Μuscle weakness may be noticed, as dantrolene’s muscle-relaxing effect works similarly on normally functioning skeletal muscles. Other side effects include drowsiness, dizziness, nausea, diarrhea, fatigue and hepatic injury [44,106].

#### 5.1.4. Opioid Antagonists

Naltrexone, an opioid antagonist, has also been examined in some studies for its potential efficacy in treating MS symptoms. It is administered orally. Low-dose naltrexone (daily doses of 1–5 mg) has been shown to reduce the proinflammatory pathway in microglia by inhibiting Toll-like receptor 4, while enhancing endogenous opioid signaling through the transient blockade of opioid receptors [107,108]. In a randomized placebo-controlled trial [109], treatment with low-dose naltrexone (4.5 mg/day for 8 weeks) was associated with self-reported improvements in mental health and pain in MS patients, with no impact on objective physical outcome measures. A 17-week randomized, double-blind, placebo-controlled trial [110] reported no statistical difference in pain, energy, emotional and physical well-being and overall quality of life in MS patients taking low-dose naltrexone compared to placebo. A retrospective chart review of 215 MS patients receiving low-dose naltrexone [111] found that 60% of the participants reported reduction in fatigue, and 75% recalled improvement in quality of life after LDN therapy. Regarding safety and tolerability, 77% of the participants reported no adverse effects. An open-label, uncontrolled trial demonstrated the potential clinical efficacy of naltrexone on physical functioning, with a significant reduction in spasticity, measured using the Modified Ashworth Scale, in 40 MS patients receiving low-dose naltrexone for 6 months [112]. However, spasticity was evaluated only as a secondary outcome in this trial. Across clinical trials, low-dose naltrexone (approximately 4.5 mg/day) has minimal side effects, such as nausea, sleep disturbance and vivid dreams or nightmares [113]. Overall, although current evidence supports the safety and tolerability of low dose-naltrexone in MS, studies examining its efficacy in MS symptoms are limited, with the majority focusing on subjective measures [113]. Further research is needed in this field to determine with sufficient evidence the therapeutic potential of naltrexone in MS.

#### 5.1.5. Botulinum Toxin Type A

Botulinum toxin type A inhibits muscle contraction by blocking the release of vesicular acetylcholine at neuromuscular junctions. It is administered through intramuscular injection. A cross-sectional study evaluated the use of BT in pain related to spasticity in 131 patients (19% of them had spasticity due to MS), with 62% reporting that botulinum toxin injections decreased their pain [114]. Two randomized placebo-controlled clinical trials assessed botulinum toxin in MS patients with severe spasticity affecting leg adductors [115,116]. In the first study [115], botulinum toxin A led to a significant reduction in spasticity (*p* = 0.009), while in the second study [116], spasm frequency was decreased in all four groups (500, 1500 or 2000 units of BT and placebo), but muscle tone was improved only in patients treated with botulinum toxin. Considering the effectiveness and side effects, the optimal dose for hip adductor spasticity was 500–1000 units, divided between the legs. Their evidence supports local injections of botulinum toxin in MS patients with spasticity of the lower limbs. A major advantage of using botulinum toxin for spasticity compared to oral muscle relaxants is the absence of central side effects. However, the most common adverse effects associated with botulinum toxin treatment are muscle weakness, fatigue, flu-like symptoms, dry mouth, dizziness and skin rash [117].

#### 5.1.6. Cannabinoids

Data indicate that cannabinoids may reduce spasticity and pain in MS patients. The activation of presynaptic CB1 receptors, predominantly expressed in the CNS, inhibits the release of neurotransmitters, leading to reduced neuronal excitability [118], while the CB2 receptors on microglia and immune cells provide anti-inflammatory and neuroprotective effects [119]. Within cannabinoids, Δ9-THC (delta-9-tetra-hydrocannabinol) is a weak partial agonist at both CB1 and CB2 receptors, with a greater affinity for CB1R [120], which modulates pain, spasticity, appetite, sedation and mood. CBD (cannabidiol) is another cannabinoid that demonstrates anticonvulsant, anti-inflammatory and analgesic effects through binding to various pharmacological targets, including CB1 and CB2 receptors (with a lower affinity than THC and a negative allosteric modulator for CB1), 5HT1a receptor, µ and δ opioid receptors, TRPV1 receptor, FAAH enzyme and PPARγ [121]. Currently, the three main medicinal cannabinoids used for the symptomatic amelioration of MS are nabiximols (oromucosal spray containing 2.7 mg of Δ9-THC and 2.5 mg of CBD/0.1 mL of solution), dronabinol (oral capsule of Δ9-THC, available in dosages of 2.5, 5 or 10 mg) and nabilone (oral capsule of 0.25, 5 or 1 mg of Δ9-THC) [122]. The medicinal cannabinoids that are licensed for MS treatment, as well as off-label use, varies widely between countries. Nabiximols are approved in many European countries and Canada as an adjunctive treatment of moderate to severe spasticity in MS [61,122]. Several trials have demonstrated the potential benefit of cannabinoids in the symptomatic treatment of MS patients. In a study evaluating 66 MS patients with central pain, THC/CBD oromucosal spray was superior to placebo in reducing mean pain intensity (mean change of −2.7, 95% CI: −3.4 to −2.0; placebo −1.4, 95% CI: −2.0 to −0.8; *p* = 0.005) [123], while in a double-blind, placebo controlled trial, dronabinol at a daily dose of 10 mg significantly reduced median spontaneous pain intensity in MS patients with central pain [124]. In a double-blind, randomized, placebo-controlled study on 160 patients, Wade et al. reported a significant difference of 22.79% (95% CI for difference = −35.52, −10.07; *p* = 0.001) on spasticity VAS scores by nabiximol in comparison with placebo [125]. Moreover, in a real-world study evaluating the efficacy and safety of nabiximols, a significant decrease in the numerical rating scale for spasticity (sNRS) and pain (pNRS) was reported, with no major adverse events [126]. A systematic review by Nielsen et al. in 2018 [127] evaluated 11 other reviews (providing data from 32 studies) on the potential benefits of cannabinoids in treating MS symptoms. Although the results for spasticity were inconsistent between studies—as many have reported positive effects on patient-rated measures of spasticity but not on the Ashworth scale—overall, most studies found evidence that cannabinoids may reduce spasticity in MS patients [127]. Moreover, a meta-analysis of 17 studies with 3161 patients in 2018 [128] assessed the efficacy and tolerability of medicinal cannabinoids in MS patients, supporting that cannabinoids can be considered a partially effective and safe therapeutic option for pain, spasticity and bladder dysfunction in pwMS. In contrast, in a randomized controlled trial on 134 MS patients in 2019–21, Hansen et al. [129] found no significant difference in the mean pain intensity (THC 0.42 (−0.54–1.38), CBD 0.45 (−0.47–1.38) and THC/CBD 0.16 (−0.75–1.08); *p* = 0.74) and mean spasticity intensity (THC 0.24 (−0.67–1.45), CBD 0.46 (−0.74–1.65) and THC/CBD 0.10 (−1.18–1.39); *p* = 0.89) between cannabis-based medicine and placebo groups. Finally, a recent systematic review and metanalysis (9 clinical trials, 2544 MS patients) reported a standardized mean difference of 39% in spasticity scores; however, the high heterogeneity and suspected bias of this study should be considered [130]. The most commonly reported adverse effects of cannabinoids are typically described as mild to moderate, including dizziness, fatigue, somnolence, vertigo, headaches, gastrointestinal symptoms, dry mouth and cognitive dysfunction [45,131]. Even though data suggest that cannabinoids may be useful for pain and spasticity in pwMS, further research is needed to evaluate the effectiveness of cannabinoids in MS symptoms.

In clinical practice, treatments for chronic pain in MS may be administered either as monotherapy or in combination, depending on the predominant pain mechanisms, symptom severity and individualized factors. Most commonly, pharmacological therapies are started as monotherapy, particularly in patients with a clearly defined phenotype. For example, neuropathic extremity pain is often initially treated either with gabapentinoids or SNRIs, while trigeminal neuralgia is typically managed with a sodium channel blocker (e.g., carbamazepine or lamotrigine) as monotherapy. Similarly, patients with nociceptive pain, which is related to spasticity, such as sustained muscle contractions and stiffness, are often initially treated with a single muscle relaxant (e.g., baclofen or tizanidine) as monotherapy.

However, in refractory cases or when mixed pain mechanisms coexist, combination therapy is not rare. Selection of the class of agent should be individualized and tailored to the specific patient’s characteristics and suspected pain mechanisms. For example, a common scenario would include a patient with chronic neuropathic pain and anxiety, depression or sleep disturbance. In this case, starting with an oral gabapentinoid, such as pregabalin or gabapentin, and adding an SNRI, such as duloxetine, would be a common approach. In the case of spasticity-related pain, the addition of muscle relaxants (e.g., baclofen or tizanidine) would be a reasonable next step. In patients with nociplastic chronic pain, antidepressants (either amitriptyline or SNRIs) represent a key therapy component, as they modulate central sensitization mechanisms through the enhancement of inhibitory pathways at the level of the raphe nuclei.

Finally, agents such as cannabinoids and botulinum toxin type A are, in general, used as add-on therapies, particularly in patients with severe spasticity, central pain refractory to first-line treatments and in patients with established central sensitization mechanisms.

Although there is not enough precise data on the frequency of combination therapy use in pwMS with chronic pain, multitherapy is commonly employed in the real-world settings, particularly in complex and treatment-resistant cases. However, caution is needed while titrating each of these agents so to avoid treatment-related adverse events, while it is essential to take into consideration the patient’s preference and comorbidities so to improve treatment adherence and reduce polypharmacy and drug-to-drug interactions.

Pharmacological treatments for chronic pain are summarized in Table 1.

**Table 1:** Pharmacological treatments of chronic pain in MS. Information summarized from the literature discussed in the main text (references [41,42,43,44,45,46,47,48,49,50,51,52,53,54,55,56,57,58,59,60,61,62,63,64,65,66,67,68,69,70,71,72,73,74,75,76,77,78,79,80,81,82,83,84,85,86,87,88,89,90,91,92,93,94,95,96,97,98,99,100,101,102,103,104,105,106,107,108,109,110,111,112,113,114,115,116,117,118,119,120,121,122,123,124,125,126,127,128,129,130,131]). The table provides a conceptual overview and is not intended as a prescribing guide.

Class	Drug	Mechanism of Action	Daily Dose
Anticonvulsants	Benzodiazepines (diazepam, clonazepam)	Increases GABA-A activity in CNS	Diazepam: 15–30 mg Clonazepam: 0.5–2 mg
	Gabapentin	Blocks α2-δ protein of voltage-gated calcium channels	900–3600 mg
	Pregabalin	Blocks α2-δ protein of voltage-gated calcium channels	150–600 mg
	Carbamazepine	Inhibits voltage-gated sodium channels	800–1200 mg
	Lamotrigine	Inhibits voltage-gated sodium channels	200–400 mg
	Levetiracetam	Binds to synaptic vesicle protein 2A (SV2A), which modulates neurotransmitter release	500–3000 mg
	Topiramate	Blocks voltage-gated sodium channels, enhances GABA-A activity, inhibits AMPA/Kainate glutamate receptors, inhibits carbonic anhydrase isoenzymes	50–300 mg
Antidepressants	Duloxetine (SNRI)	Serotonin–norepinephrine reuptake inhibitor	30–60 mg
	Amitriptyline	Inhibition of serotonin and norepinephrine transporters	10–75 mg
Cannabinoids	Nabiximols (Δ9 THC/CBD 1:1), Dronabinol (Δ9 THC), Nabilone	THC: activates CB1 and CB2 receptors CBD: low affinity for CB1 and CB2 receptors, action on 5HT1a, opioid receptors, TRPV1, FAAH, PPARγ	Nabiximols: 1–12 sprays (each spray delivers 2.7 mg ∆9-THC and 2.5 mg CBD) Dronabinol: 2.5–10 mg Nabilone: 0.25–2 mg
Muscle relaxants	Tizanidine	a2-adrenergic agonist at spinal and supraspinal levels	8–36 mg
	Oral baclofen	Pre- and postsynaptic GABA-B agonist at spinal level	30–80 mg
	Intrathecal baclofen		10–1400 mcg
	Dantrolene	Ryanodine receptor-1 antagonist, inhibits calcium release from the sarcoplasmic reticulum of the skeletal muscles	25–300 mg
Opioid receptor antagonists	Low-dose naltrexone	Toll-like receptor 4 antagonism on microglia, transient opioid receptor blockade	4.5 mg
Neurotoxin	Botulinum toxin A	Inhibits the release of acetylcholine	Typical max dose is 400 units at one site

GABA: γ-aminobutyric acid; CNS: central nervous system; SNRI: serotonin–norepinephrine reuptake inhibitor; THC: Δ9-tetrahydrocannabinol; CBD: cannabidiol; CB1/CB2: cannabinoid receptor type 1/2; 5-HT1a: serotonin 1A receptor; TRPV1: transient receptor potential vanilloid 1; FAAH: fatty acid amide hydrolase; PPARγ: peroxisome proliferator-activated receptor gamma; SV2A: synaptic vesicle protein 2A; AMPA: α-amino-3-hydroxy-5-methyl-4-isoxazolepropionic acid; α2-δ: alpha-2-delta subunit of voltage-gated calcium channels.

### 5.2. Non-Pharmacological Treatment

#### 5.2.1. Exercise

Evidence has shown that exercise interventions have beneficial effect on pain reduction in multiple sclerosis. In a systematic review and metanalysis of 10 randomized controlled trials (389 participants) [132], exercise, including aerobic, aquatic and resistance interventions, demonstrated a small to moderate beneficial effect in reducing pain associated to multiple sclerosis. Moreover, yoga and aerobic exercise improved pain, fatigue and physical and mental status in a randomized controlled study on 90 MS patients [133]. The effectiveness of yoga on the alleviation of pain symptoms in multiple sclerosis was demonstrated in another randomized clinical trial [134], in which eight 90 min yoga sessions per month in a 3-month period significantly improved VAS pain scores compared to placebo (*p* = 0.007). However, another study assessed the efficacy of yoga in MS symptoms and found no significant improvement on spasticity, fatigue and mood after 10 weeks [135]. Hydrotherapy, specifically Ai-Chi exercise (combination of deep breathing and slow, broad movements of the arms, legs and torso to work on balance, strength, relaxation, flexibility and breathing), was evaluated in a randomized controlled trial enrolling 73 MS patients [136]. After 20 weeks, Ai-Chi aquatic exercises significantly reduced pain VAS scores at a percentage of 50% (*p* < 0.028) and improved spasm, fatigue, disability and autonomy [136].

#### 5.2.2. Reflexology

Reflexology is a therapeutic approach that involves applying pressure on specific points on the feet associated with different parts of the body. A randomized, placebo-controlled trial was conducted to investigate the effectiveness of reflexology on pain in multiple sclerosis. In this study, a significant improvement in VAS pain scores (*p* < 0.001) was observed in both groups (precision and sham reflexology group) with a 50% reduction in pain levels [137], with no statistically significant difference between the two groups (*p* = 0.89). Also, a randomized controlled trial assigned 76 MS patients to three groups (reflexology, relaxation and control) and reported a significant difference in mean pain intensity in the reflexology and relaxation groups (*p* < 0.05), with the highest reduction in pain scores being observed in the reflexology group [138]. A recent systematic review and metanalysis (11 studies) evaluated the efficacy of reflexology in MS. The authors reported significant differences in pain VAS scores (−0.90, 95% confidence interval: −1.37 to −0.43) and fatigue (−1.00, 95% CI: −1.42 to −0.58) between the precision reflexology and sham reflexology groups, considering it a promising therapeutic option for pwMS [139].

#### 5.2.3. Psychological Treatments

Psychological approaches have a noteworthy contribution in pain management. Psychotherapy can target not only the pain syndrome itself but also the factors that impact it, such as sleep disturbances and mental disorders (anxiety, depression and fatigue) [89]. A Cochrane review suggested that different forms of psychotherapy had beneficial effects on pain related to multiple sclerosis, with very low level of evidence [140]. A randomized controlled trial enrolling 173 MS patients compared a telephone-delivered self-management program with the control group receiving a telephone-delivered MS educational program [141]. After 8 weeks of these interventions, 58% of patients in T-SM and 46% of patients in T-ED had 50% reduction in at least one primary outcome measure, including pain interference, fatigue and depression; however, this difference was not statistically significant. Based on within-group comparisons, T-SM participants were more activated and reported greater positive effect compared with T-ED participants, while only the T-SM group reported a decrease in pain intensity after 6 months [141]. Moreover, the effectiveness of cognitive behavioral therapy (CBT) in multiple sclerosis-related pain was evaluated in a study that used MS-related education as a comparison condition. Although there were no significant changes in pain severity, pain interference and depression severity, there was an overall improvement over time for these three outcomes in both groups, with no significant difference between them. In this study, only patients who underwent sessions of CBT had a significant progress in achieving their personally meaningful treatment goals [142]. A recent systematic review demonstrated the beneficial effects of Acceptance and Commitment Therapy (ACT) in patients with chronic pain, and noted that ACT is an effective and comparable to, if not better than, other active treatments for chronic pain [143]. A study by Harrison et al. assessed the efficacy of hybrid ACT and CBT interventions among MS patients; pain-related catastrophizing reduced in most patients, while other pain outcomes varied across individuals [144]. Moreover, in a randomized controlled trial enrolling 76 MS patients [145], Acceptance and Commitment Therapy (ACT) and Mindfulness-Based Stress Reduction (MBSR) decreased MS symptoms, including paresthesia, insomnia, fatigue and depression, and improved emotional competencies among participants. Finally, the efficacy of self-hypnosis on MS pain was evaluated in two studies. In the first study, the authors found a statistically significant pre- to post-treatment decrease in daily pain intensity (*p* < 0.001) and pain interference (*p* < 0.001) for the self-hypnosis training group [146]. In another study involving 60 MS patients [147], the mean score of pain decreased from 6.5 ± 1.8 to 3.70 ± 1.7 (*p* < 0.005) and the score for quality of pain decreased from 1.50 ± 0.47 to 0.93 ± 0.29 (*p* < 0.005) in patients performing self-hypnosis (at least 10 self-hypnosis sessions per day).

#### 5.2.4. Transcutaneous Electrical Nerve Stimulation (TENS)

TENS is widely used as a non-pharmacological approach for pain relief in various conditions. TENS is a non-invasive analgesic method that stimulates peripheral nerves by delivering electric pulses to the skin’s surface. This action modulates the transmission of nociceptive information in the CNS, thereby leading to pain alleviation [148]. A systematic review of four studies demonstrated a moderate effect of TENS for the management of central pain in patients with multiple sclerosis, despite the frequency of TENS [149]. A randomized controlled trial by Warke et al. evaluated the efficacy of TENS in low back pain in MS patients. In this study, 90 patients were randomized in high-frequency (110 Hz), low-frequency (4 Hz) and placebo groups, and were asked to self-apply TENS twice a day for 6 weeks in the form of 45 min sessions. There was a decrease in VAS scores for average low back pain over time in both the high- and low-frequency groups, but no statistically significant effect was observed [150]. In a randomized controlled trial, Miller et al. compared two weeks of 1 h and 8 h daily of TENS application at a frequency of 100 Hz in MS patients with painful spasticity. The 8 h application per day significantly reduced pain (*p* = 0.008), as measured by VAS scores, and muscle spasm (*p* = 0.038), as measured by the Penn Spasm Score, while 1 h application did not show any effect [151]. Finally, a recent randomized, controlled, single-blinded trial reported that low-frequency TENS, as well as Interferential Currents (IFCs), significantly decreased pain and increased functional capacity in MS patients [152].

#### 5.2.5. Transcranial Direct Current Stimulation (tDCS)

Noninvasive brain stimulation techniques, including transcranial direct current stimulation (tDCS), may have an analgesic effect in MS-related pain. It changes cortical excitability, notably by activating (anodal stimulation) or inhibiting (cathodal stimulation) the cortical circuits [153]. Four studies evaluated the effectiveness of tDCS in pwMS. In a randomized controlled, cross-over study, 16 MS patients were assigned to either anodal tDCS or sham tDCS groups. According to this study, the mean VAS pain score significantly decreased from 51.2 ± 19.2 at 7 days before active tDCS to 43.1 ± 26.2 at 7 days after (*p* = 0.024), and a similar improvement was noted at 1–3 days before and after each tDCS session (*p* = 0.021); no significant changes in pain scores were observed in the sham group (*p* = 0.56) [153]. Moreover, tDCS over the prefrontal cortex led to a significant amelioration in the interference subscale of BPI (Brief Pain Inventory) global score (*p* < 0.01) but not in the severity subscale. According to the authors, this could be due to the modulation of the second-order and third-order networks of the pain matrix, which are responsible for pain perception in accordance with expectations, emotions and beliefs [153]. In another study, the authors reported a statistically significant improvement in VAS pain and MPQ scores (*p* < 0.05) in MS patients with central neuropathic pain treated with anodal tDCS compared to sham tDCS. A significant improvement in quality of life was also observed among patients in the active tDCS group [154]. Additionally, a randomized controlled trial by Young et al. showed that repeated tDCS sessions for 5 days significantly reduced pain VAS scores, and this effect was maintained for up to 2 weeks post-treatment [155].

#### 5.2.6. Spinal Cord Stimulation

Spinal cord stimulation is another non-pharmacological method that has been used for the treatment of chronic pain. Currently, studies assessing the use of SCS for MS pain specifically are lacking; however, there is some evidence for its beneficial effects in multiple sclerosis. A 22-year prospective, non-controlled, observational study looked at the use of an implanted SCS system in 410 patients with chronic pain, including 19 MS patients with chronic low extremity pain. The results showed that 17 MS patients (89.5%) reported initial pain relief, and 15 patients (78.9%) continued to report good (50%) long-term pain relief in addition to improvement in gait [156]. Additionally, in two case reports, implanted SCS devices successfully reduced pain in patients with multiple sclerosis [157,158]. As for the transcutaneous use of spinal cord stimulation, in a randomized, double-blind, sham-controlled trial, MS patients treated with transcutaneous direct spinal cord stimulation had a significant improvement in neuropathic pain after 10 days of 20 min sessions, which persisted 1 month after the end of treatment [159]. In a recent small, randomized, sham-controlled trial, transcutaneous spinal cord stimulation showed a small but not significant effect on MS-related spasticity [160].

#### 5.2.7. Transcranial Random Noise Stimulation

Moreover, a randomized, sham-controlled, cross-over study investigated the efficacy of transcranial random noise stimulation, over the left dorsolateral prefrontal cortex (DLPFC), on attention and neuropathic pain in MS patients [161]. In this study, 16 MS patients randomly received two rounds of three consecutive daily sessions of either active or sham tRNS, with three weeks of wash-out interval between each round. The results demonstrated that active tRNS showed a trend to decrease N2-P2 amplitudes of pain-related evoked potentials (PREPs) and improve pain ratings (3 days before tRNS versus 3 days after tRNS, and BPI results). No significant changes for depression, anxiety and fatigue were reported. The authors suggested that this low efficacy of tRNS in pain modulation could have been more evident with longer periods of stimulation [161].

#### 5.2.8. Transcranial Magnetic Stimulation

There is a large body of evidence that transcranial magnetic stimulation can produce significant clinical improvement in various conditions, including neuropathic pain, depression and stroke [162]. Data indicate that the stimulation of the M1 region or the left dorsolateral prefrontal cortex (DLPFC) can provide an analgesic effect in patients with neuropathic pain [162,163]. As for the MS patients, most studies have examined the efficacy of repetitive transcranial magnetic stimulation on spasticity, with promising results [164,165,166]. In a review by Lefaucheur et al., the authors reported Level B evidence for intermittent theta burst stimulation (repetitive TMS protocol) targeted to the leg motor cortex for lower limb spasticity in multiple sclerosis [162]. In a study evaluating the effectiveness of rTMS on MS-related spasticity, no significant difference in pain VAS scores was reported [166], while in a study assessing the effectiveness of high-frequency rTMS (20 Hz) and iTBS (bursts at a frequency of 5 Hz), both protocols statistically significantly reduced spasticity, but only HF-rTMS led to a statistically significant decrease in pain and fatigue associated with spasticity [164].

#### 5.2.9. Neurosurgical Methods

Neurosurgical methods, such as percutaneous rhizotomy, radiosurgery techniques (Gamma-knife) and microvascular decompression, represent therapeutic options for the management of trigeminal neuralgia in pwMS. Percutaneous ganglion lesions (rhizotomies) include thermocoagulation by radiofrequency, chemical rhizotomy by glycerol injections and mechanical lesion by balloon compression [167]. These procedures could provide pain relief in patients with TN secondary to MS, but with a high risk of recurrence and often with a poorer outcome than in patients with idiopathic TN [58]. Glycerol rhizotomy has demonstrated effectiveness in the management of TN secondary to MS [167,168]. In a study by Kondziolka et al., long-term complete pain relief (mean follow-up of 36 months) was achieved in 56% of MS patients after glycerol rhizotomy, while 30% of patients required repeated procedures due to recurrence [168]. In a study by Noorani et al., glycerol rhizotomy demonstrated a shorter duration of pain relief compared to thermocoagulation and balloon compression in 33 MS patients with TN (*p* = 0.013) [169].

Radiofrequency thermocoagulation (ablation treatment) produces controlled thermal destruction of trigeminal sensory fibers, thereby interrupting the transmission of nociceptive signals [170]. In a review by Berk et al., percutaneous radiofrequency rhizotomy led to complete pain relief without the need of any medication in 81% of MS patients with medically refractory TN, though 50% experienced recurrence during a mean follow-up of 52 months [171]. Similarly, a study [172] reported complete pain relief in 82.4% of the MS patients who underwent percutaneous controlled radiofrequency rhizotomies, with 70.6% of the patients achieving this with a single procedure. A study evaluating the long-term clinical outcome of radiofrequency ablation in MS-related TN reported similar efficacy in pain outcome between initial and repeat RFT procedures at 1, 3 and 6 years (*p* = 0.77) [173]. Finally, a recent systematic review in 2025 (15 studies, 278 MS patients with TN), reported a pooled pain-free rate of 78% (95% CI: 58–93%) at the initial follow-up and 64% (95% CI: 25–95%) at the last follow-up (ranging from 17.25 to 69 months), respectively [174]. Percutaneous balloon compression is another effective and safe treatment option for TN secondary to MS [167,168,169,175,176]. This minimally invasive procedure, performed under general anesthesia, involves the insertion and inflation of a balloon into the Meckel’s cave, leading to controlled compression of trigeminal fibers and thereby the inhibition of pain transmission [177]. In a study evaluating the effectiveness of neurosurgical interventions in 96 MS patients with TN [168], balloon compression had the highest initial pain-free response and the longest duration of pain-free intervals compared to glycerol rhizotomies, stereotactic radiosurgeries, peripheral neurectomies, radiofrequency rhizotomies and microvascular decompressions. In a more recent review [178], Texakalidis et al. demonstrated no significant differences in pain outcomes between balloon compression, glycerol rhizotomy and radiofrequency ablation; however, balloon compression was associated with a higher risk of postoperative mastication weakness compared to glycerol rhizotomy (OR: 8.58; 95% CI: 1.52–48.43).

Gamma-knife radiosurgery also provides an effective alternative for the treatment of trigeminal neuralgia in pwMS. A systematic review and metanalysis (12 studies, 646 patients) reported a pooled initial pain response of 83% (CI 74–90%), decreasing to 47% (CI 33–60%), with a mean follow-up of 45 months [179]. In a recent study by Leduc et al., Gamma-knife radiosurgery showed no statistically significant difference for initial pain relief between patients with MS-related TN and idiopathic TN. However, pain recurrence occurred earlier in MS patients compared to patients with idiopathic TN (29 months vs. 75 months), and the recurrence rate was greater in TN associated with MS (78% and 52%, respectively) [180].

Finally, microvascular decompression of the trigeminal nerve root is a treatment option for the small subgroup of MS patients with no demyelinating plaques on the trigeminal nerve, but with clear neurovascular compression, demonstrated on MRI [57,181].

#### 5.2.10. Acupuncture

Acupuncture is considered a complementary and alternative medical approach and is widely used in various chronic conditions, particularly those associated with chronic stress and pain. A recent review concluded that around 14% of pwMS use acupuncture as a complementary therapy [182]. This highlights the need for well-designed studies to clarify its efficacy beyond placebo effects. Although its mechanisms of action in MS remain elusive, preclinical studies support that it exerts anti-inflammatory and neuroprotective effects through immune response modulation [182]. Early as well as more recent trials have failed to show a sustained and clinically meaningful benefit for chronic pain in pwMS [182]. Therefore, current evidence does not support its role in chronic pain management in pwMS and more studies with robust methodologies are required to clarify whether any therapeutic benefit exists.

Non-pharmacological treatments for chronic pain are summarized in Table 2.

**Table 2:** Non-pharmacological treatments of chronic pain in MS.

Intervention	Study Design/Evidence	Main Outcomes on Pain	Limitations/Comments
Exercise (aerobic, resistance, aquatic, yoga)	Systematic reviews and RCTs	Small to moderate pain reduction; improvements in fatigue, physical and mental status	Heterogeneous protocols; mixed results across studies
Hydrotherapy (Ai-Chi)	RCT	Significant reduction in pain (≈50%), spasm, fatigue and disability	Limited number of studies
Reflexology	RCTs; systematic review and meta-analysis	Pain and fatigue reduction; no superiority over sham in some trials	Strong placebo effect; methodological variability
Psychological interventions (CBT, ACT, MBSR, self-management)	RCTs; systematic reviews	Improvement in pain interference, coping and goal attainment; inconsistent effects on pain severity	Low certainty of evidence; variable outcome measures
Self-hypnosis	Small clinical trials	Significant reduction in pain intensity and interference	Small sample sizes; limited replication
TENS	RCTs; systematic reviews	Moderate effect on central pain; benefit dependent on stimulation duration	Inconsistent results; protocol variability
tDCS	RCTs	Reduction in pain intensity and pain interference; improved quality of life	Small samples; short follow-up
Transcranial random noise stimulation (tRNS)	RCT	Trend toward pain reduction; no significant clinical effects	Possibly insufficient stimulation duration
Repetitive TMS	RCTs; reviews	Effective for spasticity-related pain; inconsistent effect on pain alone	Pain often secondary outcome
Spinal cord stimulation (implanted/transcutaneous)	Observational studies; RCTs; case reports	Sustained pain relief in selected patients	Invasive (implanted); limited MS-specific trials
Neurosurgical procedures (TN-specific)	Observational studies; systematic reviews	High initial pain relief in MS-related trigeminal neuralgia	High recurrence rates; risk of complications
Acupuncture	RCTs; systematic reviews	No sustained or clinically meaningful benefit for chronic pain	Strong placebo effect; insufficient high-quality trials

ACT: Acceptance and Commitment Therapy; Ai-Chi: aquatic exercise combining deep breathing and slow movements; CBT: cognitive behavioral therapy; MBSR: Mindfulness-Based Stress Reduction; MS: multiple sclerosis; RCT: randomized controlled trial; TENS: transcutaneous electrical nerve stimulation; tDCS: transcranial direct current stimulation; tRNS: transcranial random noise stimulation; TN: trigeminal neuralgia;.

## 6. Discussion

Although highly prevalent and disabling, chronic pain remains underrecognized and undertreated in pwMS. It has major impact on the quality of life of patients and affects functional outcomes. Epidemiological studies have come up with widely varied prevalence, which reflects methodological discrepancies across them. However, chronic pain is experienced by nearly two-thirds of pwMS at some point during their disease course.

Pathophysiologically, chronic pain involves various mechanisms, including nociceptive, neuropathic and mixed or nociplastic components. It may manifest with several different clinical syndromes, such as trigeminal neuralgia, migraine, spasticity-related pain or extremity pain. Each condition has specific clinical and pathophysiological characteristics and a respective treatment approach. Psychological and social factors, such as catastrophizing, along with negative expectations, such as nocebo phenomena, as well as social isolation can all contribute to pain chronicity through central sensitization and pain perception modulation

An important implication of this present review is that chronic pain should not be seen as a single clinical entity but rather as a spectrum of overlapping clinical phenotypes. These phenotypes are guided by distinct, interactive pathophysiological mechanisms which may dynamically evolve over the disease course, contributing to pain chronicity and treatment resistance. The modest efficacy of some first-line agents may partly be explained by this mechanistic overlap and underscores the importance of mechanism-based treatment selection approach. Instead of focusing on pain location and intensity, comprehensive evaluation and diagnosis of specific pain phenotype based on our suggested classification may result in more efficacious treatment selections and sequencing.

Heterogeneous mechanisms correspond to different treatments; hence, a wide arsenal of symptomatic pain therapies is available for pwMS. Options include anticonvulsants such as benzodiazepines, pregabalin, gabapentin, lamotrigine, topiramate and other antiepileptics. Antidepressants, including duloxetine or amitriptyline, have been successfully used and constitute a significant point of treatment, particularly for neuropathic pain. Other treatments include opioid antagonists as well as cannabinoids.

Although a wide range of pharmacological treatments for chronic pain in MS exists, there is a lack of high-quality evidence supporting many commonly used agents. Most of the treatments are redirected from studies in other conditions with neuropathic pain, while MS-specific studies investigating chronic pain are extremely limited and methodologically heterogeneous. Furthermore, in most cases, their efficacy is modest, while treatment related side effects such as cognitive slowing and neuropsychiatric symptoms are common and particularly disabling in the already vulnerable MS population. These limitations highlight the need for a careful, mechanism-based and patient-centered treatment selection, combining pharmacological agents only in refractory cases to avoid polypharmacy and the risk of drug interactions.

Besides pharmacological treatments, non-pharmacological approaches have increasingly been used over the last decades with varied levels of success. These include cognitive behavioral therapy, reflexology, physiotherapy and, most importantly, exercise, which undoubtedly remains one of the unexploited disease modifying therapies in MS.

Although these methods are commonly employed as adjunctive therapy modalities, the overall quality of evidence supporting their efficacy for chronic pain remains limited. Most available studies are small, heterogeneous in design, with predominantly subjective outcomes and highly susceptible to placebo responses. Consequently, complementary and alternative medicine approaches should be adjunctive rather than evidence-based primary treatments. For the time being, their role is to support pharmacological treatment and their use should be guided by patients’ preference and, most importantly, safety considerations.

Future research in MS-related chronic pain should move toward a more integrated and mechanism-driven framework. Prospective studies with precise pain phenotyping, neuroimaging markers and patient-reported outcomes are needed to better understand and manage chronic pain in MS. Central sensitization and dysfunction of central pain pathways should be taken into consideration when assessing pain phenotypes, as nociplastic mechanisms are underrecognized yet common in pwMS. Combination therapies need to be evaluated systematically in randomized clinical trials, as evidence is still limited. Moving forward, personalized treatment strategies should be prioritized and explored based on specific patients’ characteristic, comorbidities and other concomitant medication.

In conclusion, pain is a prevalent and disabling symptom resulting from complex interactions between demyelinating lesions, maladaptive neuroplasticity and psychosocial factors in pwMS. Effective management requires an individualized, mechanism-based approach which should combine pharmacological and non-pharmacological modalities. Advancing research into further understanding the neurobiological aspects and treatment optimization of MS-related pain remains a critical priority for improving patient quality of life and functional outcomes.

## Data Availability

No new data were created or analyzed in this study. Data sharing is not applicable to this article.

## Abbreviations

The following abbreviations are used in this manuscript:

5-HT1A	serotonin 1A receptor
Δ9-THC	delta-9-tetrahydrocannabinol
ACT	Acceptance and Commitment Therapy
AMPA	α-amino-3-hydroxy-5-methyl-4-isoxazolepropionic acid
BT	botulinum toxin
BPI	Brief Pain Inventory
CB1	cannabinoid receptor type 1
CB1R	cannabinoid receptor type 1 receptor
CB2	cannabinoid receptor type 2
CBT	cognitive behavioral therapy
CBD	cannabidiol
CGRP	calcitonin gene-related peptide
CI	confidence interval
CNS	central nervous system
DLPFC	dorsolateral prefrontal cortex
DRESS	Drug Reaction with Eosinophilia and Systemic Symptoms
FAAH	fatty acid amide hydrolase
GABA	gamma-aminobutyric acid
GABA-A	gamma-aminobutyric acid type A receptor
GABA-B	gamma-aminobutyric acid type B receptor
IASP	International Association for the Study of Pain
IFCs	interferential currents
iTBS	intermittent theta burst stimulation
ITB	intrathecal baclofen
LDN	low-dose naltrexone
MBSR	Mindfulness-Based Stress Reduction
MPQ	McGill Pain Questionnaire
MRI	magnetic resonance imaging
MS	multiple sclerosis
NNT	number needed to treat
OR	odds ratio
PAG	periaqueductal gray
pNRS	pain numerical rating scale
PPARγ	peroxisome proliferator-activated receptor gamma
pwMS	people with multiple sclerosis
PPMS	primary progressive multiple sclerosis
RFT	radiofrequency thermocoagulation
RRMS	relapsing-remitting multiple sclerosis
rTMS	repetitive transcranial magnetic stimulation
SCS	spinal cord stimulation
SJS	Stevens–Johnson syndrome
sNRS	spasticity numerical rating scale
SNRI	serotonin–norepinephrine reuptake inhibitor
SPMS	secondary progressive multiple sclerosis
SV2A	synaptic vesicle protein 2A
TENS	transcutaneous electrical nerve stimulation
tDCS	transcranial direct current stimulation
TN	trigeminal neuralgia
TRPV1	transient receptor potential vanilloid 1
tRNS	transcranial random noise stimulation
VAS	visual analog scale
